# Development of a New Vaccine for the Prevention of Lassa Fever

**DOI:** 10.1371/journal.pmed.0020183

**Published:** 2005-06-28

**Authors:** Thomas W Geisbert, Steven Jones, Elizabeth A Fritz, Amy C Shurtleff, Joan B Geisbert, Ryan Liebscher, Allen Grolla, Ute Ströher, Lisa Fernando, Kathleen M Daddario, Mary C Guttieri, Bianca R Mothé, Tom Larsen, Lisa E Hensley, Peter B Jahrling, Heinz Feldmann

**Affiliations:** **1**Virology Division, United States Army Medical Research Institute of Infectious DiseasesFort Detrick, MarylandUnited States of America; **2**Department of Pathology, Uniformed Services University of the Health SciencesBethesda, MarylandUnited States of America; **3**Special Pathogens Program, National Microbiology LaboratoryPublic Health Agency of Canada, Winnipeg, ManitobaCanada; **4**Department of Immunology, University of ManitobaWinnipeg, ManitobaCanada; **5**Department of Medical Microbiology, University of ManitobaWinnipeg, ManitobaCanada; **6**Department of Biological Sciences, California State UniversitySan Marcos, CaliforniaUnited States of America; **7**Pathology Division, United States Army Medical Research Institute of Infectious DiseasesFort Detrick, MarylandUnited States of America; **8**Headquarters, United States Army Medical Research Institute of Infectious DiseasesFort Detrick, MarylandUnited States of America; University of BonnGermany

## Abstract

**Background:**

Recent importation of Lassa fever into Germany, the Netherlands, the United Kingdom, and the United States by travelers on commercial airlines from Africa underscores the public health challenge of emerging viruses. Currently, there are no licensed vaccines for Lassa fever, and no experimental vaccine has completely protected nonhuman primates against a lethal challenge.

**Methods and Findings:**

We developed a replication-competent vaccine against Lassa virus based on attenuated recombinant vesicular stomatitis virus vectors expressing the Lassa viral glycoprotein. A single intramuscular vaccination of the Lassa vaccine elicited a protective immune response in nonhuman primates against a lethal Lassa virus challenge. Vaccine shedding was not detected in the monkeys, and none of the animals developed fever or other symptoms of illness associated with vaccination. The Lassa vaccine induced strong humoral and cellular immune responses in the four vaccinated and challenged monkeys. Despite a transient Lassa viremia in vaccinated animals 7 d after challenge, the vaccinated animals showed no evidence of clinical disease. In contrast, the two control animals developed severe symptoms including rashes, facial edema, and elevated liver enzymes, and ultimately succumbed to the Lassa infection.

**Conclusion:**

Our data suggest that the Lassa vaccine candidate based on recombinant vesicular stomatitis virus is safe and highly efficacious in a relevant animal model that faithfully reproduces human disease.

## Abstract 2

A replication-competent vaccine based on attenuated recombinant vesicular stomatitis virus vector protects non- human primates.

## Introduction

Among the viruses causing severe hemorrhagic fever in Africa, Lassa fever is a significant public health problem in Nigeria, Liberia, Sierra Leone, and the Republic of Guinea. It is estimated that Lassa virus infects over 200,000 individuals per year across this region, causing over 3,000 deaths [1]. The case fatality rate for Lassa fever is around 15% in hospitalized patients and has been greater than 50% in several outbreaks [2,3]. Human infection is associated with contact with a widely distributed and highly commensal rodent, *Mastomys natalensis,* or by contact with infected patients. Recent importation of Lassa fever into Germany, the Netherlands, the United Kingdom, and the United States by travelers on commercial airlines [4–8] illustrates the potential for the spread of this highly dangerous and contagious pathogen. In addition, Lassa virus has gained notoriety because it is classified as a Category A bioweapon agent [9].

Lassa virus is an enveloped, bisegmented RNA virus belonging to the Old World group within the family Arenaviridae [10]. Arenavirus particles contain a genome consisting of two ambisense single-stranded RNA molecules, designated small (S) and large (L), of a length about 3.4 kb and 7.2 kb, respectively. The S segment contains two genes that encode three structural proteins, the nucleoprotein (NP) and the envelope glycoproteins GP1 and GP2. The L segment contains two genes that encode two proteins, the viral polymerase (L protein) and the Z protein. NP and L protein associate with the genomic RNA in a ribonucleoprotein complex or nucleocapsid structure. It is thought that the Z protein functions as a matrix protein and is responsible for the formation of virus particles [11]. GP1 and GP2 are initially synthesized as a precursor molecule, glycoprotein C (GPC), which is posttranslationally cleaved by the protease SKI-1/S1P [12]. GP1 is the portion of the surface glycoprotein spike that is thought to be the effector for receptor binding, while GP2 is structurally consistent with viral transmembrane fusion proteins of other enveloped viruses [13].

Currently, there are no vaccines or antiviral drugs approved for Lassa fever. Treatment with intravenous ribavirin was shown to reduce mortality from Lassa fever in high-risk patients and presumably decreases morbidity in all patients with Lassa fever [14]. However, the availability of ribavirin is very limited in endemic areas, and treatment is most effective if initiated within the first week of disease onset [14]. Preventing contact with the reservoir host in endemic areas is currently unachievable; therefore, a preventive vaccine is a critical public health need, especially to protect health care providers, who are often the most at risk. Indeed, the recent untimely death of a well-known doctor in Sierra Leone who treated thousands of cases of Lassa fever and ultimately contracted the disease as a result of these laudable efforts [15] underscores the need for a preventive vaccine.

Early attempts to develop a vaccine against Lassa fever focused on classical approaches such as killed vaccines. A whole-virion vaccine inactivated by gamma irradiation provided a good humoral response to Lassa viral proteins, NP, and GP, but failed to protect nonhuman primates from a lethal Lassa challenge [16]. As these animals were unprotected despite a strong humoral response, it was suggested that protection would depend on a robust cellular response. Subsequent efforts to develop an efficacious vaccine against Lassa fever focused on genetically engineered vaccines. Specifically, studies focused on recombinant vaccinia viruses expressing the NP, the full-length GPC, and combinations of GP1 and GP2. In a series of studies by Fisher-Hoch and colleagues, approximately 90% of nonhuman primates that received a vaccine containing both GP1 and GP2 survived a lethal Lassa challenge [1,17,18]. Protection did not correlate with humoral immunity, as none of these animals had demonstrable Lassa virus-specific antibody before challenge. Consequently, cell-mediated immunity was implicated in protecting these animals, although T-cell responses were not measured in these studies. In comparison, vaccination of macaques with NP alone resulted in the development of relatively high antibody titers before Lassa challenge, but only 20% survival.

While the recombinant vaccinia vaccines expressing the full-length Lassa GPC protected nearly 90% of nonhuman primates from a lethal challenge, it was noted that the vaccinia platform is not suitable for human use because of potential side effects, which is a particular concern in areas where HIV prevalence is high [1]. However, it appears from all previous studies that a strong cellular response may be required for protection. Vaccination with a live virus characteristically elicits strong cellular immune responses, and live vaccines are particularly attractive because they can confer protection in a single injection. Therefore, the ideal Lassa vaccine would be based on a replication-competent format engineered for enhanced safety and with the ability to overcome technical obstacles such as prior immunity to the vector. Recently, we described the generation of a live attenuated, recombinant vesicular stomatitis virus (rVSV) expressing the GPC of Lassa virus, strain Josiah [19]. Vaccines based on live attenuated rVSV have been highly effective in animal models and are particularly attractive because they can be administered by the mucosal route [20–23]. Furthermore, vesicular stomatitis virus (VSV) infections in humans occur fairly rarely worldwide, mainly in the enzootic regions of the Americas, and consequently, global pre-existing immunity is negligible [24]. Because the guinea pig model of Lassa fever does not faithfully mimic human disease [25] and is not generally predictive for efficacy of vaccines and antivirals [1], we evaluated the protective efficacy of the replicating rVSV vector in nonhuman primates.

## Methods

### Vaccine Vectors and Viruses

**Figure 1:**
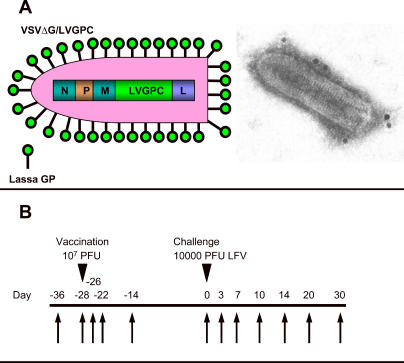
Lassa Virus Vaccine Design and Vaccination Regimen (A) Left, schematic diagram of the recombinant VSV expressing the glycoprotein of Lassa virus. The VSV glycoprotein (G) was replaced with the Lassa virus glycoprotein (LV GPC). Shown are the nucleocapsid (N), phosphoprotein (P), matrix (M), and RNA-dependent RNA polymerase (L). Right, gold sphere labeling (10-nm spheres) of VSVΔG/LVGPC particles by immunoelectron microscopy using a pool of two murine monoclonal antibodies directed against Lassa virus GP1 and GP2; particles were negatively contrasted with 1% uranyl acetate. (B) Time line for vaccination and Lassa viral challenge study. Vaccination of cynomolgus monkeys was done with a single intramuscular dose of 10^ 7^ PFU of either VSVΔG/LVGPC (four animals) or VSVΔG/ZEBOVGP (two animals). Challenge was performed with a single intramuscular dose of 10^4^ PFU of Lassa virus, strain Josiah. Arrows indicate day of sampling (blood and swabs).

The recombinant VSV expressing the glycoprotein of Lassa virus, strain Josiah, and Zaire ebolavirus (ZEBOV), strain Mayinga, were generated as described recently using the infectious clone for the VSV, Indiana serotype (kindly provided by John Rose, Yale University, New Haven, Connecticut, United States) [19]. Briefly, the appropriate open reading frames for the glycoproteins were generated by PCR, cloned into the VSV genomic vectors lacking the VSV glycoprotein gene, sequenced-confirmed, and rescued using the method described earlier [26]. The recombinant viruses expressing Lassa virus glycoprotein and ZEBOV glycoprotein were designated VSVΔG/LVGPC (Figure 1A) and VSVΔG/ZEBOVGP, respectively. Cynomolgus macaques were challenged with Lassa virus isolated from a human case in 1976 in Sierra Leone [27].

### Animal Studies

Six cynomolgus macaques *(Macaca fascicularis),* 4–6 y old and weighing between 3 kg and 8 kg, were obtained from approved suppliers. Four of the animals were vaccinated intramuscularly with approximately 2 × 10^7^ PFU of VSVΔG/LVGPC, and two with an equivalent dose of VSVΔG/ZEBOGP (controls). The six cynomolgus macaques were challenged intramuscularly 28 d after the single-dose immunization with 1 × 10^4^ plaque-forming units (PFU) of Lassa virus, Josiah strain. Animals were closely observed twice daily after the immunization and the challenge for clinical symptoms. Swab samples (oral, nasal, rectal, vaginal) and blood were collected as shown in Figure 1B. Studies were performed in biosafety level (BSL)-4 biocontainment. Research was conducted in compliance with the Animal Welfare Act and other federal statues and regulations relating to animals and experiments involving animals, and adheres to the National Research Council laboratory animal care principles [28]. The facility used is fully accredited by the Association for Assessment and Accreditation of Laboratory Animal Care International.

### Hematology and Serum Biochemistry

Total white blood cell counts, white blood cell differentials, red blood cell counts, platelet counts, hematocrit values, total hemoglobin, mean cell volume, mean corpuscular volume, and mean corpuscular hemoglobin concentration were determined from blood samples collected in tubes containing EDTA, by using a laser-based hematologic analyzer (Coulter Electronics, Hialeah, Florida, United States). The white blood cell differentials were performed manually on Wright-stained blood smears. Serum samples were tested for concentrations of albumin (ALB), amylase, alanine aminotransferase (ALT), aspartate aminotransferase (AST), alkaline phosphatase (ALP), gamma-glutamyltransferase (GGT), glucose, cholesterol, total protein, total bilirubin (TBIL), urea nitrogen (BUN), and creatinine (CRE) by using a Piccolo Point-Of-Care Blood Analyzer (Abaxis, Sunnyvale, California, United States).

### Virus Detection

RNA was isolated from blood and swabs using the appropriate RNA isolation kits from QIAGEN (Mississauga, Ontario, Canada). To detect VSV we used an RT-PCR assay targeting the matrix gene. Lassa viral RNA was detected using a primer pair targeting the gene encoding L protein. Virus was titrated by plaque assay on Vero E6 cells from all blood and selected and swab samples and from tissue samples collected at necropsy (liver, spleen, lung, kidney, adrenal gland, pancreas, lymph nodes, bone marrow, ovary, testis, and brain) [29].

### Humoral Immune Responses

IgG antibodies against Lassa virus were detected with an ELISA using purified viral particles as an antigen source [30]. Neutralization assays were performed by measuring plaque reduction in a constant virus:serum dilution format as previously described [29]. Briefly, a standard amount of Lassa virus (approximately 100 PFU) was incubated with serial 2-fold dilutions of the serum sample for 60 min. The mixture was used to inoculate Vero E6 cells for 60 min. Cells were overlaid with an agar medium and incubated for 8 d. Plaques were counted 48 h after neutral red staining. Endpoint titers were determined by the dilution of serum which neutralized 50% of the plaques.

### Cellular Immune Responses

Peripheral blood mononuclear cells (PBMCs) were isolated by histopaque gradient (Sigma, St. Louis, Missouri, United States) from each animal before vaccination and transformed with Herpesvirus papio to serve as antigen-presenting cells. Each day on which assays were performed, 5 × 10^5^ transformed cells from each animal were dispensed and infected with VSVΔG/LVGPC at a multiplicity of infection of 1.0. At 12 h postinfection, transformed cells from each animal were individually mixed with PBMCs collected from the respective animals. For example, transformed cells from subject 1 were mixed with PBMCs collected from subject 1. Anti-CD28 and anti-CD49d antibodies (1.0 μg of each antibody; BD Pharmingen, San Diego, California, United States) and GolgiPlug (BD Pharmingen) were added to each coculture and incubated for 6 h at 37 °C. Staphylococcal enterotoxin B was added as a positive control to one set of cultures. Cultures were placed at 4 °C overnight. Cocultured cells were stained for CD8, CD3, and CD4 in Pharmingen stain buffer (BD Pharmingen) for 20 min at 4 °C in the dark. Cells were washed with Pharmingen stain buffer and fixed/permeabilized with Cytofix/Cytoperm buffer (BD Pharmingen) for 1–1.5 h in the dark at 4 °C. Cells were then washed in 1× Perm/Wash buffer (BD Pharmingen) and stained with interferon gamma (IFN-γ)-allophycocyanin or tumor necrosis factor alpha (TNF-α)-allophycocyanin in 1× Perm/Wash buffer for 45 min at 4 °C in the dark. Cells were next washed in 1× Perm/Wash buffer and resuspended in Cytofix/Cytoperm buffer for analysis. Acquisition was performed on a FACS Calibur flow cytometer collecting 100,000 to 200,000 lymphocyte-gated events per sample, and analyzed using FloJo software (Tree Star, Ashland, Oregon, United States). Cytokine-positive cells were defined as a percentage of double-positive cells for CD8 and CD4 populations within the lymphocyte gate.

### Statistics

Vaccine efficacy was calculated using Fisher's exact test. A *p*-value of 0.05 or lower was considered significant.

## Results

### Protection against Lassa Fever

We tested the Lassa fever rVSV vaccine candidate in nonhuman primates. Four cynomolgus macaques *(M. fascicularis)* were immunized by intramuscular injection with a single dose of approximately 2 × 10^7^ PFU of VSVΔG/LVGPC, and two control animals received VSVΔG/ZEBOVGP (same route and dose). The animals were monitored closely for clinical symptoms and shedding of rVSVs. Figure 1B shows the inoculation and sampling protocol that was followed for this study. After vaccination, none of the nonhuman primates displayed any signs of clinical symptoms, indicating that the rVSVs were apathogenic for these animals. All six animals were subsequently challenged intramuscularly on day 28 postvaccination with a high dose (1 × 10^4^ PFU) of Lassa virus. The two control animals (vaccinated with irrelevant VSVΔG/ZEBOVGP) started to show clinical signs of illness on day 3, when one of the animals had a fever (defined as a temperature over 104 °F). By day 10, both control animals developed macular rashes and anorexia, and one animal had severe facial edema, which is prognostic for a poor outcome in humans [31]. These control animals succumbed to the Lassa virus challenge and were euthanized on day 11 and day 13, respectively. At necropsy, both controls showed lesions and pathological changes consistent with Lassa fever in nonhuman primates [25,32]. In contrast, none of the VSVΔG/LVGPC-vaccinated animals became sick, and all four animals were fully protected against the high Lassa challenge dose. Survival was statistically significant (*p* = 0.0079 by Fisher's exact test) when the four VSVΔG/LVGPC-vaccinated macaques were compared to the two nonspecifically vaccinated animals and three recent controls that succumbed to a Lassa challenge using the same dose of the same virus isolate delivered by the same route (T. W. G., unpublished data).

**Figure 2:**
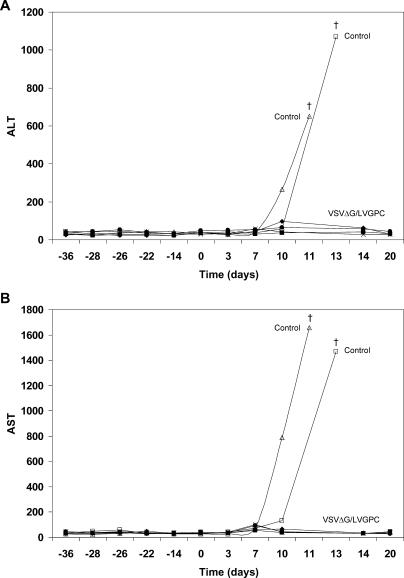
Hepatic Enzyme Levels in Sera of Cynomolgus Monkeys After Challenge with Lassa Virus Results of ALT (A) and AST (B) assays are shown for the four VSVΔG/LVGPC-vaccinated macaques (closed symbols) and the two control monkeys (open symbols).

Analysis of blood chemistry and hematology was performed on the indicated days (Figure 1B; Table 1); values in the VSVΔG/LVGPC-vaccinated animals did not differ significantly from pre-challenge values. There were slight perturbations in platelet counts at day 7 after Lassa challenge in three of the four VSVΔG/LVGPC-vaccinated animals, with a small decrease in platelet numbers. This modest drop in platelets was also noted in one of the two VSVΔG/ZEBOVGP-vaccinated control animals at day 7. In addition, a marked lymphopenia developed in both control animals, but was not observed in any of the four VSVΔG/LVGPC-vaccinated macaques. Significant changes in clinical chemistry (greater than 3-fold changes from baseline values) were seen in both control animals in ALT and AST (Figure 2). In addition, increases were also seen in serum levels of ALP, GGT, TBIL, and BUN, while decreased levels of ALB and CRE were evident in one of these animals. The only perturbation in clinical chemistry values among the four VSVΔG/LVGPC-vaccinated animals was very slight increases in the ALT levels in two of the animals at day 7 and a third monkey at day 10.

**Table 1:**
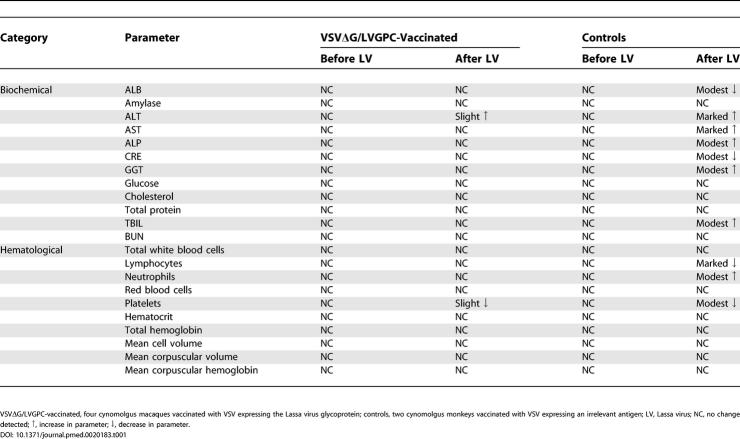
Blood Chemistry and Hematology Results for Cynomolgus Monkeys before and after Challenge with Lassa Virus

VSVΔG/LVGPC-vaccinated, four cynomolgus macaques vaccinated with VSV expressing the Lassa virus glycoprotein; controls, two cynomolgus monkeys vaccinated with VSV expressing an irrelevant antigen; LV, Lassa virus; NC, no change detected; ↑, increase in parameter; ↓, decrease in parameter.

DOI: 10.1371/journal.pmed.0020183.t001

### No Viremia or Shedding of Vaccine Vector

**Figure 3:**
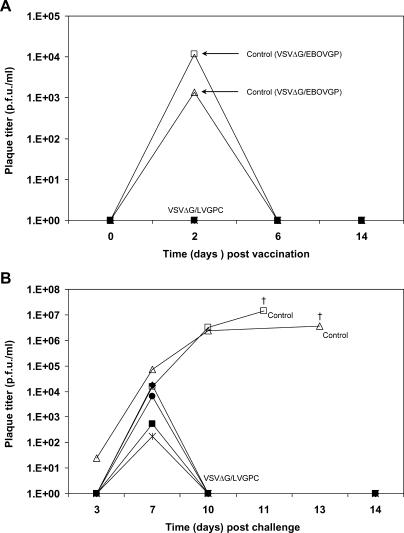
Viremia in Nonhuman Primates after Vaccination and Lassa Virus Challenge Viremia levels after vaccination (A) and Lassa virus challenge (B) were determined in plasma taken at the indicated times after vaccination and Lassa virus challenge (see Figure 1B). RNA was isolated from plasma, and PCR specific for VSV and Lassa virus were performed as described in Methods. Note: Subjects 1–4 were vaccinated with VSVΔG/LVGPC, while controls 1 and 2 were vaccinated with VSVΔG/ZEBOVGP.

To determine if viremia or shedding of the rVSVs occurs after vaccination, whole blood and swab samples (oral, nasal, vaginal, and rectal) from all six vaccinated animals were analyzed by virus isolation and RT-PCR assay. A moderate viremia was detected on day 2 postimmunization by virus isolation (10^3^–10^4^ PFU/ml) and RT-PCR in the two VSVΔG/ZEBOVGP-vaccinated control animals, but viremia was not detected at any time point in any of the four VSVΔG/LVGPC-vaccinated macaques (Figure 3A). No evidence of shedding was detected by virus isolation or RT-PCR in any of the swab samples from any of the six animals evaluated. Thus, there is no evidence that virus could be shed under the conditions tested.

### Vaccine Protects against Lassa Fever but Immunity Is Not Sterile

Lassa viral replication and shedding was analyzed by virus isolation and RT-PCR from the blood and swab samples taken after Lassa challenge (see Figure 1B). Organ samples from the two control animals (vaccinated with irrelevant VSVΔG/ZEBOVGP) were also available. At day 3 after Lassa challenge, viremia was detected in one of the two control animals (Figure 3B), but not in the second control animal or in any of the four VSVΔG/LVGPC-vaccinated monkeys. By day 7, all six monkeys were viremic (viremia up to approximately 10^4^ PFU/ml) (Figure 3B). However by day 10, all four of the VSVΔG/LVGPC-vaccinated animals had cleared the viremia, while both control animals had high viremias (more than 10^6^ PFU/ml), which was maintained until euthanasia. High levels of Lassa virus were also detected in organ samples of these two control animals (10^4^–10^9^ PFU/g) (unpublished data).

### Strong Humoral and Cellular Immune Response after Lassa Virus Challenge

**Figure 4:**
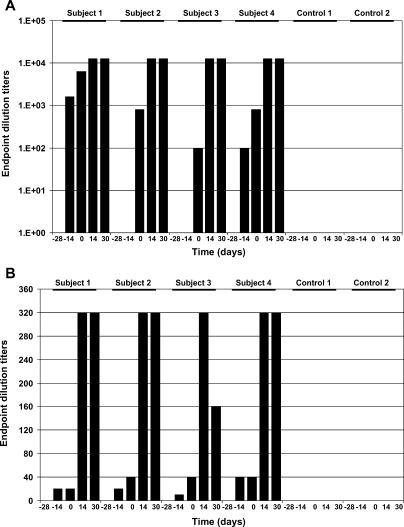
Humoral Immune Response in Nonhuman Primates to Lassa Virus before and after Lassa Challenge (A) IgG responses were measured using an established ELISA (see Methods). The titers are presented as endpoint dilutions. (B) Neutralizing antibodies were detected by a plaque reduction neutralization assay as described in Methods. Titers are presented as endpoint dilutions. Note: Subjects 1–4 were vaccinated with VSVΔG/LVGPC, while controls 1 and 2 were vaccinated with VSVΔG/ZEBOVGP.

**Figure 5:**
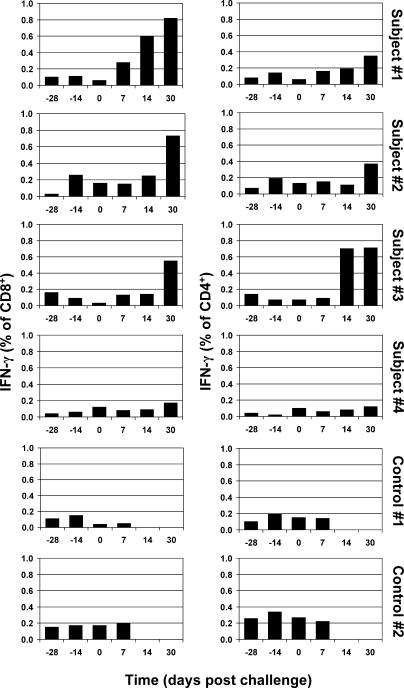
Cellular Immune Response (IFN-γ) in Nonhuman Primates before Vaccination, after Vaccination, and after Lassa Virus Challenge Intracellular levels of IFN-γ were determined in CD4- and CD8-positive T-cell populations before vaccination (−28), after vaccination (−14 and 0), and after challenge with Lassa virus (7, 14, and 30); challenge was administered on day 0. Strong cellular responses following restimulation with transformed cells expressing Lassa GPC were seen in three of the four animals after challenge. Note: Subjects 1–4 were vaccinated with VSVΔG/LVGPC, while controls 1 and 2 were vaccinated with VSVΔG/ZEBOVGP.

**Figure 6:**
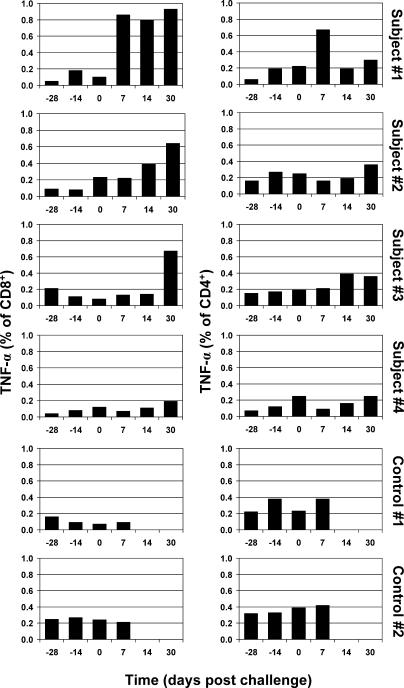
Cellular Immune Response (TNF-α) in Nonhuman Primates before Vaccination, after Vaccination, and after Lassa Virus Challenge Intracellular levels of TNF-α were determined in CD4- and CD8-positive T-cell populations before vaccination (−28), after vaccination (−14 and 0), and after challenge with Lassa virus (7, 14, and 30); challenge was administered on day 0. Strong cellular responses following restimulation with transformed cells expressing Lassa GPC were seen in three of the four animals after challenge. Note: Subjects 1–4 were vaccinated with VSVΔG/LVGPC, while controls 1 and 2 were vaccinated with VSVΔG/ZEBOVGP.

By the day of Lassa challenge, all four VSVΔG/LVGPC-vaccinated monkeys had developed moderate- to high-level IgG antibody titers (Figure 4A), with three of four animals having titers over 1 in 800. In addition, all four VSVΔG/LVGPC-vaccinated monkeys developed low-level neutralizing antibody titers against Lassa virus (Figure 4B). After Lassa virus challenge, ELISA and neutralizing antibody titers markedly increased in all four VSVΔG/LVGPC-vaccinated monkeys (Figure 4). The cellular immune response was measured by intracellular cytokine staining for IFN-γ and TNF-α in CD4- and CD8-positive lymphocytes. Before Lassa virus challenge, the production of IFN-γ and TNF-α was detectable in CD8-positive lymphocytes in one of four VSVΔG/LVGPC-vaccinated monkeys (subject 2). After Lassa virus challenge, three of the four VSVΔG/LVGPC-vaccinated animals responded positively, with values between 0.15% to 0.93% positive IFN-γ- or TNF-α-positive CD8 cells (Figures 5 and 6). Two of these three animals also ranged between 0.19% and 0.36% positive IFN-γ- or TNF-α-positive CD4 cells. These results indicate that the VSVΔG/LVGPC was a potent stimulator of humoral and cellular immunity.

## Discussion

The VSV-based vector expressing the Lassa virus GPC mediated complete protection of four of four cynomolgus monkeys from a high-dose lethal challenge of Lassa virus. Protection was associated with the generation of Lassa-specific CD8^+^ T cell and antibody responses. Although the presence of preexisting ELISA and neutralizing anti-GP antibodies were associated with protection, previous studies in nonhuman primates showed little to no anti-glycoprotein antibodies after vaccination with vaccinia recombinants expressing the full-length Lassa viral glycoprotein [18]. Notably, significant increases in the levels of antibodies were detected after the vaccinia-immunized macaques were challenged with infectious Lassa virus, but levels of neutralizing antibodies were not observed before or after Lassa virus challenge in these studies. Indeed, in the current study, we also observed increased levels of antibodies in the VSVΔG/LVGPC-vaccinated animals after Lassa virus challenge, including substantial increases in the levels of neutralizing antibodies. While previous studies have implied that cellular immunity is necessary for protection against Lassa fever, no previous study to our knowledge has evaluated the cellular immune response in nonhuman primates. Here, we show that cytotoxic T lymphocytes appear to play an important role in protection, as production of IFN-γ and TNF-α was detected in CD8-positive lymphocytes in three of the four VSVΔG/LVGPC-vaccinated macaques after Lassa challenge. However, we cannot discount the importance of neutralizing antibodies as another potential mechanism of protection.

In this study, protection against Lassa fever was dependent on vaccination with an attenuated, replication-competent virus, which may raise questions regarding the safety of live-attenuated vectors. Most importantly, we demonstrated here that, despite a short-term viremia in two animals immunized with the control VSVΔG/ZEBOVGP vaccine, rVSV replication and shedding were not detectable in nonhuman primates, and the animals did not develop fever or other symptoms, nor were there changes in blood chemistry or hematology.

Increased concern about natural or artificial introductions of emerging viruses such as Lassa virus has driven increased investment in basic research and construction of a network of biocontainment laboratories. In the past, a relatively small global market and a lack of biocontainment facilities generated little commercial interest for developing a Lassa fever vaccine. Therefore, there have been relatively few attempts to evaluate vaccines against Lassa fever in nonhuman primate models. It is difficult to compare results across these few studies because of a number of variables, including differences among animal species and dose, route, and strain of challenge virus employed. It is important to note that two different species of nonhuman primates *(M. fascicularis* and *M. mulatta)* were employed in the previous studies evaluating vaccinia virus as a platform for a Lassa fever vaccine [1,17,18]. Those studies, which used seven cynomolgus monkeys, offer a good comparison with the current study, as these animals were immunized with a vaccinia construct expressing the Lassa viral GPC and were challenged with 10^4^ PFU of Lassa virus (Josiah strain) [18]. Five of these seven vaccinia-Lassa GPC-vaccinated cynomolgus monkeys were protected from a lethal Lassa viral challenge, compared to the result of protection of four of four cynomolgus monkeys from 10^4^ PFU of Lassa virus (Josiah strain) in the current study. Differences between these two vaccine platforms were also noted in circulating Lassa viral loads and clinical illness. While Lassa viremia was detected in both studies in all vaccinated animals at postinfection day 7, we failed to detect viremia in any VSVΔG/LVGPC-vaccinated animal at day 10. In comparison, in the previous study, which employed the vaccinia virus platform, viremia was detected in four of the seven vaccinated animals at day 10 [18]. Consistent with differences in the duration of viremia, we failed to detect any evidence of clinical illness in any of the four VSVΔG/LVGPC-vaccinated macaques after Lassa virus challenge, while a transient febrile illness with moderate physiological changes was reported in the vaccinia-Lassa GPC-vaccinated monkeys after Lassa virus challenge [1]. All experimental controls died in the current study and the previous studies [1,18]. The Lassa viral challenge seed used in our study was uniformly lethal in 12 of 12 cynomolgus monkeys challenged by the same route with a comparable dose [33] and three of three cynomolgus monkeys with the exact same dose (T.W.G., unpublished data); thus, protection of four of four VSVΔG/LVGPC-vaccinated monkeys is highly significant (*p* = 0.0079).

It will be important to determine whether this rVSV vaccine can protect against other strains of Lassa virus or whether successful vaccination will require additional genes to confer heterologous protection. Others have demonstrated that vaccination of nonhuman primates with the nonpathogenic and closely related Mopeia virus protects animals from a lethal Lassa virus (Josiah strain) challenge [17,18] suggesting that broad protection is an attainable goal.

The potency of the immune responses induced in this study led us to speculate that complete protection could be achievable in less than the 28 d between immunization and challenge, but this notion, of course, must be experimentally tested. A shortened regimen would be a significant advance in the event of either a natural outbreak or bioterrorism, as both occur rapidly with little warning and require swift and effective measures to reduce their impact on risk groups such as health-care and military personnel. An immediate response is needed to contain an outbreak and prevent the spread of disease to other geographic regions. So far, quarantine practices appear to be effective in limiting Lassa outbreaks, which have been most severe in rural hospitals [2,3]. However, infection and mortality have been devastating in the quarantined community [2,3], and modern advances in global travel cannot ensure that future outbreaks will be as easily contained. While rodent control would be an effective way to greatly reduce the number of cases of Lassa fever in West Africa, current economic barriers make such control unlikely. Thus, the availability of a vaccine that could be rapidly employed to create a ring of vaccination around an epidemic zone would be critical to controlling the subsequent spread of Lassa virus. Here, our one-dose, 4-wk, rVSV-based Lassa immunization regimen demonstrates the plausibility of developing a candidate vaccine to meet this need.

The recent focus on development of vaccines against highly pathogenic viruses has been concentrated on various recombinant vectors (e.g., adenoviruses, alphavirus replicons, vaccinia, DNA vaccination) for expression of virus-encoded proteins in various combinations to induce protective immunity [29,34,35]. While many of these approaches show promise, an important technical obstacle that is inherent with most of these platforms is the potential for prior immunity to the vector either through natural exposure or prior immunization [36–38]. This is an area in which the rVSV platform has a competitive advantage over many of the other formats. Preexisting immunity in the human population against VSV is minimal and may not be important at all, because neutralizing antibodies are directed against the VSV glycoprotein [21], which is not expressed using the recombinant vector described here.

The primary concern regarding use of the rVSV vaccine platform in humans is related to the fact that this is a replication-competent vaccine, and thus demonstration of safety is of paramount importance. Pathogenicity associated with VSV and rVSVs is a concern that will require further investigation. However, the VSV glycoprotein, which is thought to be the main determinant of pathogenicity for VSV, has been completely deleted in the VSV platform that we describe for Lassa virus. In fact, it has been shown that mutations truncating the VSV glycoprotein cytoplasmic domain from 29 to nine or one amino acid are nonpathogenic in mice [21,39], and, not unexpectedly, there is an absence of vector-associated pathogenesis in mice receiving vectors with complete deletion of the VSV glycoprotein [39]. Reassortment and recombination are other concerns that have been associated with the use of replication-competent vaccine platforms. Importantly, the VSV single-stranded RNA genome does not undergo reassortment and therefore lacks the potential of reassorting with wild-type viruses in vivo. Furthermore, VSV replicates within the cytoplasm of infected cells and does not undergo genetic recombination.

Durability is an important concern and a desirable trait of any vaccine platform. Indeed, this is an even more important consideration when developing vaccines against exotic pathogens such as Lassa virus that exist in remote geographic locales where boosting is often problematic or not feasible at all. In these settings, the ideal vaccine would be a single-shot vaccine capable of providing protection for life or for a very long period of time. Replication-defective vaccines typically do not provide such durability. Live viral vaccines have traditionally offered the most effective long-term protection against viral infections. Such vaccines tend to induce strong cellular and humoral host immune responses as a result of the intracellular synthesis of specific antigens at high levels over a prolonged period. Thus, in addition to low seroprevalence in humans, durability is another important advantage of exploiting VSV as a vaccine platform. While safety remains a hurdle that must be overcome before the potential of this platform can be fully realized, a number of groups are currently working toward developing the rVSV vaccine platform for eventual use in humans. Research with rVSV-based vaccines is underway for HIV, cancer therapy, and other applications. Notably, the National Institute of Allergy and Infectious Disease recently awarded a 5-y, 22.8-million dollar contract to Wyeth to expand research on a candidate rVSV-based HIV vaccine that was shown to prevent AIDS-like disease in monkeys [40]. Indeed, the use of replicating rVSV-based vectors has proven to be a potent and promising concept for future vaccine development against Lassa fever and perhaps other lethal viral hemorrhagic fever agents.

**Patient Summary**

**Background**

Lassa fever is a disease caused by a virus that is often spread by rodents. The disease is common in parts of West Africa where it causes a significant amount of death and disability among the population. Recently, Lassa fever has been imported by travelers to the United States and Europe. The Lassa virus that causes the disease is also on the list of potential bioweapons agents.

**Why Was This Study Done?**

A vaccine against the Lassa virus could help to control the disease in Africa, protect health workers, and help contain viral outbreaks. Several groups had developed different vaccines, some of which could protect monkeys from getting sick. However, none of the vaccines developed so far have shown all the characteristics one needs to have before testing them in humans.

**What Did the Researchers Do?**

Many vaccines combine specific parts of the harmful (pathogenic) virus with other components from harmless viruses, resulting in an effective but safe vaccine. The researchers developed a vaccine using a virus called vesicular stomatitis virus as the harmless component (known as the carrier). They inserted some genetic material from the harmful Lassa virus. In their study, they gave four macaque monkeys one shot of this combination vaccine and two others shots of the “empty” carrier (i.e., just the harmless component).

**What Did They Find?**

When four weeks later they injected all six monkeys with a lethal dose of Lassa virus, the four monkeys injected with the combination vaccine stayed healthy. The two monkeys who had been given just the “empty” carrier died.

**What Does This Mean?**

These are promising early results that justify further testing of this particular vaccine.

**What Next?**

Issues that need to be resolved before the vaccine can be tested in humans include the safety of the vesicular stomatitis carrier virus, how long the vaccine protects after the shot, and whether it is active against different strains of the Lassa virus (there are at least four strains, each with slightly different genetic material).

**More Information Online**

Information on Lassa fever can be found at the following Web sites.

The US CDC Special Pathogens Branch Web site: http://www.cdc.gov/ncidod/dvrd/spb/

The WHO fact sheet on Lassa Fever (from 2000): http://www.who.int/mediacentre/factsheets/fs179/en/

## Abbreviations

ALB: albumin
ALP: alkaline phosphatase
ALT: alanine aminotransferase
AST: aspartate aminotransferase
BUN: urea nitrogen
CRE: creatinine
GP: glycoprotein
GPC: glycoprotein C
IFN-γ: interferon gamma
NP: nucleoprotein
PBMC: peripheral blood mononuclear cell
PFU: plaque-forming unit(s)
TBIL: total bilirubin
TNF-α: tumor necrosis factor alpha
rVSV: recombinant vesicular stomatitis virus
VSV: vesicular stomatitis virus
